# Visualization of system dynamics using phasegrams

**DOI:** 10.1098/rsif.2013.0288

**Published:** 2013-08-06

**Authors:** Christian T. Herbst, Hanspeter Herzel, Jan G. Švec, Megan T. Wyman, W. Tecumseh Fitch

**Affiliations:** 1 Department of Cognitive Biology, Laboratory of Bioacoustics, University of Vienna, Althanstrasse 14, 1090 Vienna, Austria; 2 Institute for Theoretical Biology, Humboldt University Berlin, Invalidenstrasse 43, 10115 Berlin, Germany; 3 Department of Biophysics, Faculty of Science, Palacký University Olomouc, 17 Listopadu 12, 771 46 Olomouc, CzechRepublic; 4 Mammal Vocal Communication and Cognition Research Group, School of Psychology, University of Sussex, Pevensey Building, Falmer BN1 9QH, UK

**Keywords:** phasegram, system dynamics, sliding-window analysis, attractor visualization, Poincaré section, bioacoustics

## Abstract

A new tool for visualization and analysis of system dynamics is introduced: the *phasegram*. Its application is illustrated with both classical nonlinear systems (logistic map and Lorenz system) and with biological voice signals. Phasegrams combine the advantages of sliding-window analysis (such as the spectrogram) with well-established visualization techniques from the domain of nonlinear dynamics. In a phasegram, time is mapped onto the *x*-axis, and various vibratory regimes, such as periodic oscillation, subharmonics or chaos, are identified within the generated graph by the number and stability of horizontal lines. A phasegram can be interpreted as a bifurcation diagram in time. In contrast to other analysis techniques, it can be automatically constructed from time-series data alone: no additional system parameter needs to be known. Phasegrams show great potential for signal classification and can act as the quantitative basis for further analysis of oscillating systems in many scientific fields, such as physics (particularly acoustics), biology or medicine.

## 1. Introduction

Oscillating systems, either forced or self-sustaining, are found in many branches of physical or biological science. They range from simple harmonic oscillators to complex nonlinear systems. Particularly in signals (i.e. time-series data) from biological systems, three principal modes of operation are frequently observed: periodic oscillation, subharmonics and chaos [1–4].^1^

In order to assess the complexity of a dynamic system, several analysis parameters have been developed, such as the correlation dimension [5] or the Lyapunov exponent [6]. These measures work well on stationary signals^2^ (where system parameters are not varying in time), but are less well equipped to deal with signals where system parameters change—as for most (bio)physical data [7,8]. To cater for these needs, analysis can be performed with sliding windows (i.e. the progressional analysis of shorter portions of the signal, extracted at consecutive time instants).

In many applications, the spectrogram is the de facto standard for continuous windowed analysis of periodicity and/or the dynamic evolution of (bio)physical time-series data. A spectrogram is a type of sliding power spectral analysis. When created with a narrow-band setting (resulting in a high-frequency resolution), the (dis)appearance of spectral peaks in time, owing to bifurcations occurring in the underlying system, provides certain important insights into the system's dynamics [8,9].

While the spectrogram is an analysis tool that is easily accessible to a broad range of researchers, it does not provide direct information on the system's dynamics in phase space, i.e. a mathematical space where all possible states of a system are represented (see Nolte [10] for an essay on the history of phase space). In phase space, each possible state of the system corresponds to one unique point. Temporal developments of the system are delineated by the so-called trajectories within the phase space. For mechanical systems, each degree of freedom (d.f.) of the system is mapped onto an individual dimension in *n*-dimensional phase space.

In the following paragraphs, we will show the advantages and disadvantages of the discussed visualization methods, i.e. sliding-window analysis (represented by the spectrogram) and phase space diagrams.

**Figure 1.**
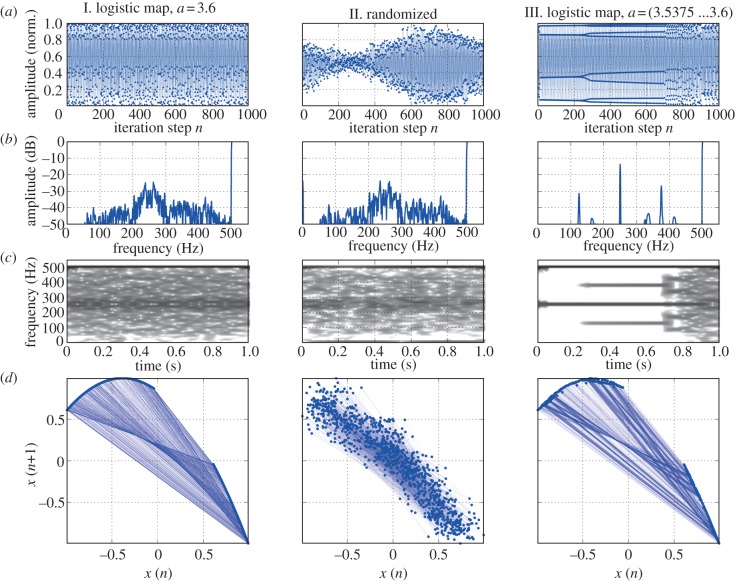
Illustration of limitations of both spectrograms and phase space diagrams. Left column: I. deterministic chaos, created by a logistic map; middle column: II. stochastic signal created by additive synthesis with randomized phases; right column: III. signal created by a logistic map where the parameter *a* was gradually varied from 3.5375 to 3.6. (*a*) All 1000 data points of each time series. (*b*) Amplitude spectrum. (*c*) Spectrogram. (*d*) Phase space embedding. Note that both signals I. and II. look identical when analysed by Fourier series (*b*) and (*c*), whereas their different nature becomes apparent in phase space (*d*). The additional complexity of signal III. (period doubling cascade to chaos) is not apparent in the phase space diagram (*d*).

The spectrogram's inability to distinguish deterministic chaos from a random process is illustrated in figure 1. A synthesized signal was generated from a logistic map with parameter *a* set to a value of 3.6 (please refer to case 1, later in the text, for a definition of the logistic map) at a sampling rate of 1000 Hz. The resulting irregular time-series data are shown in the left column of figure 1*a* (signal I). The signal's spectrum is displayed in figure 1*b*, and a spectrogram is shown in figure 1*c*. The phase space diagram^3^ (figure 1*d*) reveals that the system's trajectory is aligned along a parabola [4, p. 357].

The middle panel (II) of figure 1 shows raw and analysis data of a series of superimposed sine waves with randomized phase offsets, whose amplitudes are derived from the spectral analysis of the logistic map signal (I). Although signals I and II are virtually indistinguishable by spectral analysis (figure 1*b*,*c*, left and middle column), the phase space for signal II contains no pronounced attractor but a random distribution, revealing the stochastic nature of signal II.

In order to illustrate the limitation of phase space diagrams, a signal exhibiting a series of bifurcations has been created from the logistic map, by gradually varying parameter *a* from 3.5375 to 3.6 (signal III in figure 1, right column). The development of the period doubling cascade is clearly visible in the spectrogram (being a sliding-window analysis that is capable of visualizing temporal developments), but the fact is obscured in the phase space diagram (figure 1*d*, right column), where all of the different regimes are superimposed.

Non-stationary signals, potentially exhibiting multiple bifurcations over the course of a recorded time series, occur frequently in the (bio)physical domain, and they are particularly common in acoustical data. Here, we address the need for an intuitive visualization tool that displays the time-varying dynamics of these systems. We introduce a method that combines the advantages of both sliding-window analysis (i.e. the sensitivity to temporal developments in a signal) and phase space diagrams (i.e. their close relation to the system's underlying dynamics). This new tool, termed the ‘phasegram’, is able to visualize system dynamics over time in a single two-dimensional graph. The usefulness of this method will be exemplified for the specific field of voice science using a series of examples with different levels of complexity and control over the underlying system.

## 2. Methods

### 2.1. Phasegram generation

**Figure 2.**
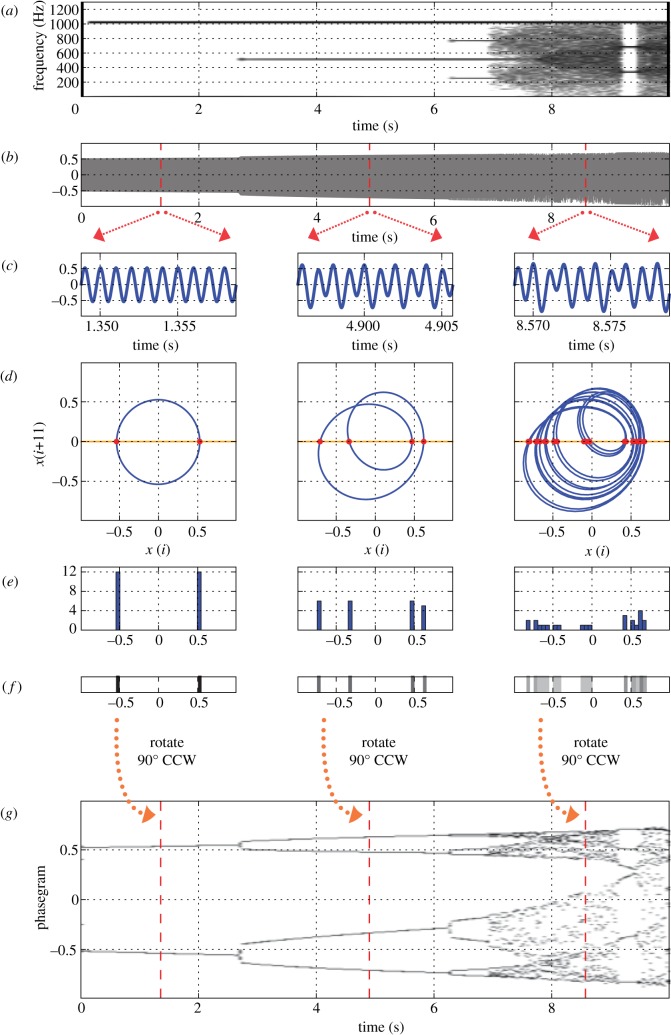
Process of phasegram generation. (*a*) Spectrogram of a signal synthesized from the logistic map (see text). (*b*) Time-domain signal of *x*[*i*]. (*c*) Three portions of the signal, extracted at times *t* = 1.35, *t* = 4.9 and *t* = 8.57 s. (*d*) Phase portraits from the above signals, created by attractor reconstruction. A Poincaré section was created along the *x*-axis (orange), yielding intersection points with the trajectory (red dots). (*e*) Histograms of trajectory intersection points with Poincaré sections for all three extracted signal portions. For better visibility, a very large histogram bin width of 0.025 was chosen. (*f*) ‘Trajectory strips’: colour-coded histograms of Poincaré sections through phase portraits (see text); (*g*) phasegram from the signal displayed in (*a*) and (*b*). The markers at *t* = 1.35, *t* = 4.9 and *t* = 8.57 s represent the position of the three trajectory strips from (*f*) within the graph.

The phasegram generation process is analogous to creating electroglottographic (EGG) wavegrams, a method previously developed by Herbst *et al*. [11]. Phasegram generation is outlined below (see also figure 2), and will be described in more detail in the following paragraphs.

— Generation of two-dimensional phase portraits, extracted from consecutive windows in time (optional: creation of a so-called phase portrait movie).
— Creation of Poincaré sections through the two-dimensional phase portraits.
— Conversion of Poincaré section crossings into trajectory histograms.
— Conversion of trajectory histograms into ‘trajectory strips’ by colour-coding.
— Combination of rotated ‘trajectory strips’ into the final phasegram.

### 2.2. Step 1: generation of phase portraits, extracted at consecutive points in time

A phase portrait is the geometrical representation of the trajectories of a dynamical system, providing a description of the system's evolution in time [9]. If the governing differential equations of a dynamical system are not known (the typical case when analysing a biological system), then the dynamics of the phase space can be analysed via attractor reconstruction [12,13]: the attractor is defined as a set on the phase plane to which all neighbouring trajectories converge [4]. In attractor reconstruction, a two-dimensional vector2.1
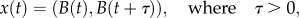
is defined, based on the analysed signal. The time series *B*(*t*) appears as a trajectory *x*(*t*) in a two-dimensional phase space [4, p. 438]; in other words, the signal is projected against a delayed version of itself (see the electronic supplementary material, figure S1). This time delay **τ** should typically be in the range of one-tenth to one-half the mean orbital period around the attractor [4, p. 440]. A suitable value for **τ** is typically found when the analysed signal's autocorrelation function first passes through zero.

An alternative method for determining the ideal value for **τ** has been proposed by Frazer & Swinney [14], who suggest considering the first minimum in the mutual information function of the attractor as the proper time delay **τ**. This approach should ensure the minimized redundancy of information between the embedding axes.

As a third option, the analysed time-series data can be converted into an analytic signal by means of a Hilbert transformation. The Hilbert transform shifts the phase of each negative frequency component of the analysed signal by +90° (**π**/2 radians) and the phase of the positive frequency components by −90° (−**π**/2 radians). For a purely sinusoidal signal that contains only one frequency component, for instance, applying the Hilbert transform for phase portrait generation would have the same effect as using a lag of one quarter of the sine wave's period. In order to create a phase portrait from Hilbert-transformed data, the real values of the analytic signal are plotted against the imaginary values [15]. This approach is particularly useful for longer signals with either highly variable cycles or without apparent fundamental frequency.

While attractor reconstruction can theoretically be performed in an unlimited number of dimensions, two dimensions are chosen for practical reasons for the purpose of phasegram generation. In a discrete-time (i.e. sampled) signal, equation (2.1) becomes2.2
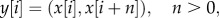
where *i* is the sample index and *n* is the number of samples delay between the two versions of the signal. The process of phase portrait generation is further detailed in the electronic supplementary material.

For the creation of a phasegram, a series of phase portraits is required. These are constructed for consecutive portions of the analysed signal, centred at constantly progressing time instants. These portions are rectangular windows of the signal, defined as [*x*[*i − m*] … *x*[*i* + *m*]], where *m* is half the window length, and *i* is the sample index around which the window is centred. For the consecutive generation of multiple phase portraits, *i* is advanced by steps of *f*_S_**τ**, where *f*_S_ is the sampling frequency and **τ** is the time step (usually in the range of 0.01–0.02 s).

For optimal phasegram output, any DC offset should be removed from the analysed time-series data before phase portrait generation.^4^ In extreme cases where longer signals exhibit a considerable baseline drift, a phase-preserving high-pass filter with a cut-off frequency of 1 or 2 Hz can be applied.

As an optional visualization tool, a ‘phase portrait movie’ may be created, by converting the phase portraits (extracted at time instants increasing by 0.04 s, which corresponds to the default video frame rate of 25 fps) to a video file. The original signal can be included as an audio channel, by converting the signal to an audio file (WAV file type) with a sampling frequency of *F*_S_ = 1/d*t* (Hz), where d*t* (given in seconds) is the time interval at which the original time-series data were sampled. The synchronous playback of both the auditory stimulus and time-varying phase portraits provides valuable insights into the analysed sequence. It can also serve as the basis upon which the manual rotation of Poincaré sections (see below) can be performed, if so desired. The phase portrait movies for the examples discussed in this manuscript are available as electronic supplementary material.

### 2.3. Step 2: creation of Poincaré sections through the two-dimensional phase portraits

A Poincaré section is the intersection of a dynamic system's trajectory in the phase space with a certain lower dimensional subspace [9, p. 64]. In a system with *n*-dimensions, the Poincaré section is an (*n−*1)-dimensional surface of section [4, p. 278]. Hence, the Poincaré section of a two-dimensional phase portrait, as created by means of attractor reconstruction, is a (one-dimensional) line.

The Poincaré section generation process is illustrated in figure 2*d*: a line is drawn at a certain angle (horizontal in the case of figure 2*d*) through the two-dimensional phase portrait. The number and location of intersections of the phase space trajectory and the line (the red dots in figure 2*d*) are determined and stored for further analysis. For the purpose of phasegram generation, the Poincaré section is made through the entire phase portrait (see the electronic supplementary material, movie S1 and the audio track contained therein for an illustration of the principle).

The default orientation of the Poincaré sections through the phase portraits is either horizontal or vertical. In some cases, these angles cannot capture crucial aspects of the emerging attractor. In such cases, the angle of the Poincaré sections (i.e. the lines drawn across the phase portraits) needs to be adjusted. An optimal angle can be determined visually by inspection of the phase portrait movies. Alternatively, a fully automated autonomous algorithm to determine the rotation angle of the Poincaré section can be used, which is described in the electronic supplementary material. This algorithm aims to find the Poincaré sections that are best suited to reveal the full complexity of the analysed signal's attractor. The phase portrait angles of the examples shown in the subsequent case studies were all determined with the suggested algorithm.

**Figure 3.**
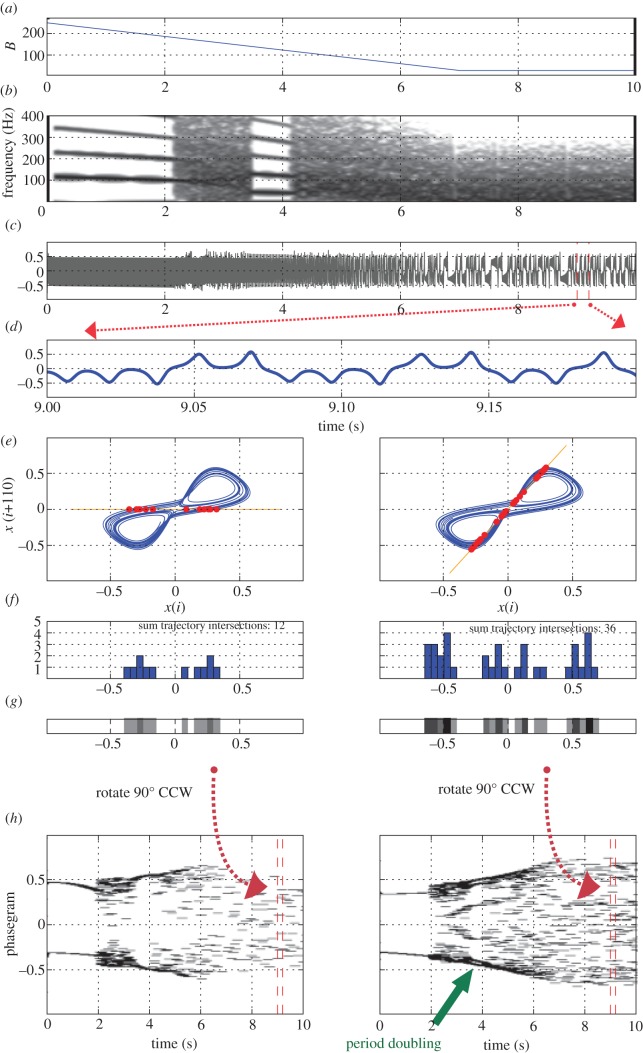
Effect of the Poincaré section angle on phasegram generation, illustrated with a Lorenz system. (*a*) Parameter *B* of the Lorenz system, varied from 250 to 28 (see text). (*b*) Spectrogram of generated time-domain signal (output variable *x* of the Lorenz system). (*c*) Time-domain signal. (*d*) A 200 ms portion of the time-domain signal, centred around *t* = 9 s. (*e*) Phase portraits from the above signals, created by attractor reconstruction. A Poincaré section was created along the *x*-axis (left panel) and at an angle of 0.35 π radians, as determined by the algorithm described in the electronic supplementary material (right panel), yielding intersection points with the trajectory (red dots). (*f*) Histograms of the trajectory intersection points on Poincaré sections in the two conditions. (*g*) ‘Trajectory strips’: colour-coded histograms with Poincaré sections through phase portraits. (*h*) Phasegrams from the signal displayed in panel (*c*) for both Poincaré section angles, respectively. The markers at *t* = 9 s represent the position of the trajectory strips from (*g*) within the graph. See text for the note on the period doubling sequence at *t* = 3.4–3.8 s in the right panel.

The effect of the Poincaré section angle in phasegram generation is exemplified in figure 3 by a signal generated with a Lorenz system [16, equations 25–27]—for details see below. Poincaré sections were created at 0° (left panel) and 45° (right panel). The effect of the angle variation is seen in the histograms, the trajectory strips and the resulting phasegrams (figure 3*f–h*). Note that the phasegram created with the algorithmic angle selection (right panel in figure 3*h*) reveals a subharmonic sequence around *t* = 3.4–3.8 s, which is not apparent in the phasegram created with an arbitrary angle of 0 radians (left panel in figure 3*h*)—see also electronic supplementary material, movie S2.

### 2.4. Step 3: conversion of Poincaré sections into trajectory histograms

For each phase portrait, a histogram of the trajectory intersection points is generated (figure 2*e*). The histogram bin width is dependent on the overall signal amplitude, and on the graph height of the resulting phasegram. For the phasegrams presented in this manuscript, values in the range of 0.001–0.01 were used for signals that were normalized to [−1 … 1].

### 2.5. Step 4: conversion of trajectory histograms into ‘trajectory strips’ by colour-coding

The maximum number of trajectory intersection points (hist_max_) within one single histogram bin from the Poincaré section histograms is determined for all analysed phase portraits. All Poincaré section histograms are then converted (flattened) to ‘trajectory strips’, i.e. they are colour-coded by2.3
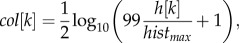
where *col*[*k*] is the colour intensity (ranging from 0 to 1) and *h*[*k*] is the number of trajectories found in the *k*th histogram bin (see figure 2*f* for examples).

### 2.6. Step 5: concatenation of rotated ‘trajectory strips’ into a phasegram

All resulting colour-coded ‘trajectory strips’ are rotated anticlockwise by 90°, and concatenated in sequence to form the phasegram (see figure 2*g*, where the process is exemplified by three out of 500 total trajectory strips). In the phasegram, time is displayed on the *x*-axis, representing the instants in time at which the individual phase portraits have been created (algorithm step 1). The *y*-axis corresponds to the bin index of the Poincaré section histogram. The colour intensity shows the frequency of occurrence of phase portrait trajectory intersections with the Poincaré section, as seen in the respective histograms.

For the purpose of this manuscript, phasegram generation was performed using custom software^5^ written by author C.T.H. in Python using the matplotlib library [17], and the phase portrait movies were created from successive still images with the free software ffmepg [18].

### 2.7. Analysed scenarios

In order to illustrate their applications for yielding insights into time-varying biological signals, phasegrams have been generated for five scenarios of increasing complexity. The first two cases are general systems with well-known dynamics. The third case is a computational simulation of vocal fold vibration. The last two cases were generated from real signals: the first of these was created with an excised larynx set-up in which all the crucial underlying parameters are experimentally controlled. In the other example, a human singing signal, the oscillating system is only controlled in a subjective arbitrary manner by the singer (‘increasing and decreasing the intensity, trying to avoid abrupt changes in sound colour’).

## 3. Results

### 3.1. Case 1: logistic map. Period doubling cascade

A synthetic signal was created from the logistic map:3.1
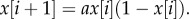


Equation (3.1) was evaluated for values of *a* in the range of [3.4 … 3.65], and the change of the parameter *a* was mapped linearly onto a time interval of 10 s with a sampling frequency of 1000 Hz. The resulting signal was then up-sampled to 44100 Hz with the software Praat [19]. This processing step introduced additional data points into the time series by means of band-limited interpolation using a sinc function kernel (sinc(*x*) = sin(π *x*)/(π *x*); see [20]), thus allowing display of the logistic map time series as limit cycles in the phase portraits (see step 1 and figure 2*c*,*d*). The DC offset was removed from the resulting time series data by simple subtraction of the mean, and the signal was normalized to [−1 … 1].

The relationship between a portion of the time-domain signal, its phase portrait and its visual appearance in the phasegram is clearly seen in figure 2*c,d* and *g*.

— For a sine wave, i.e. the simplest form of periodic vibration, the phase portrait is a simple limit cycle, having the form of a circle or ellipse—left panel in figure 2*d*. Because the signal is perfectly periodic (figure 2*c*), only two intersection points are seen in the phase portrait, even though the Poincaré section is intersected by the trajectory 12 times in each direction. This is reflected in the histogram (figure 2*e*), where a total of 24 intersections are distributed over only two histogram bins. Consequently, only two horizontal lines are seen in the phasegram in the case of periodic oscillation/the limit cycle (figure 2*g*, *t* = 0–2.66 s).
— In the period doubling case (centre column of panels in figure 2), the phase portrait trajectory must complete two revolutions in the limit cycle before repeating itself. Because there are a total of four intersection points along the Poincaré section, the intersections (a total of 23) are distributed over four histogram bins (middle panel in figure 1*e*). Consequently, four horizontal lines are seen in the phasegram in the interval of *t* = 2.6–6.23 s in figure 2*g*.
— The third column of panels in figure 2 exemplifies the case of a non-periodic signal: a complex pattern is seen in the phase portrait. A total of 24 intersection points along the Poincaré section are distributed over a large number (20) of histogram bins. Because the signal is non-periodic at *t* = 6.9–9.1 s and *t* = 9.4–10 s, the histogram bin maxima vary greatly over time, and the phasegram obtains a ‘rugged’ appearance at these time intervals in figure 2*g*.

The resulting phasegram in figure 2*g* has a striking similarity to the standard bifurcation diagram of the logistic map. The difference is that the phasegram is derived from real data, without knowledge of the underlying time-varying parameter.

### 3.2. Case 2: Lorenz attractor. Chaos

The Lorenz system [16, equations 25–27] is defined as:3.2
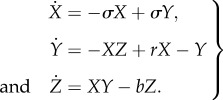


This system was transformed into a set of difference equations:3.3
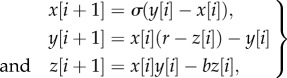
and evaluated for a duration of 10 s. The simulation was run with a time step of 1/882 s, and a sped-up version of the resulting time-series data was then saved with a sampling rate of 44 100 Hz. The parameter *r* was varied gradually from 250 to 28 over an interval of 7 s, and then kept stable at a value of 28 for another 3 s (figure 3*a*). The initial values were set to *x* = 0, *y* = 20, *z* = 25. The vector *x*[*i*]*, i* = [0 … 441 000] was further analysed.

When simulating the Lorenz system (see equations (3.2) and (3.3)), parameter **σ** is usually fixed at 10, and *b* is defined to be 8/3 [16]. Setting *r* to a value of 28 results in the well-known ‘strange attractor’ of the Lorenz system. A higher value of *r* (above 30) can lead to both stable oscillatory regimes and some areas of period doubling bifurcations. This is reflected in the phasegram in the right panel of figure 3*h*: periodic and stable from *t* ≈ 0 to 2 s; period doubling from *t* ≈ 3.5 to 4 s; chaotic from *t* ≈ 4 to 10 s. The chaotic nature of the system for a stable value of *r* (*t* = 7–10 s) is revealed by the phasegram in figure 3*h* (right panel): the intersection points of the phase portraits extracted at various instants within this interval vary unpredictably, but stay within the regions defined by the strange attractor emerging in the phase portrait (see figure 3*e*).

### 3.3. Case 3: self-oscillating two-mass model of the vocal folds

In most mammals (including humans), voice signals are generated by the flow-induced, self-sustaining vibration of laryngeal tissue [21]. The steady air stream coming from the lungs is converted into a time-varying airflow by the oscillation of the laryngeal tissue (mainly the vocal folds). The pressure variations caused by the time-varying airflow are then propagated through, and acoustically filtered by, the vocal tract. Finally, the result of this process is radiated from the mouth (and/or nose) as an acoustical signal [22]. Voice is a widely researched physical system that can exhibit a great variety of oscillatory behaviour, such as periodic vibration, subharmonics, chaos and bifurcations between any of these phenomena [3,23–27].

**Figure 4.**
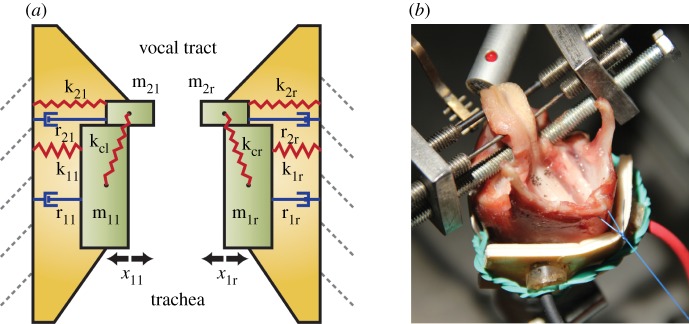
(*a*) Schematic of the two-mass model of the vocal folds. Each vocal fold is approximated by two coupled oscillators. The springs and dampers represent the viscoelastic properties of the vocal folds. The time-varying lateral displacements *x*1l and *x*1*r* were taken as the input to phasegram analysis. (*b*) Excised larynx set-up: sika deer larynx mounted on an air supply tube, EGG electrodes attached on thyroid cartilage at level of vocal folds.

In previous research, a simplified two-mass model of voice production was created in order to study the effect of asymmetries on vocal fold vibration [28]. In this model, each vocal fold is represented by two coupled oscillators (defined by their mass, stiffness and damping coefficients; figure 4*a*). This model provides 2 d.f. per vocal fold. It allows for the two masses of each vocal fold to oscillate with a phase difference (the lower mass typically leading the vibration), thus capturing the most essential mechanism of self-sustaining vocal fold vibration: the transfer of aerodynamic energy into tissue vibrations [29]. The model has the option of simulating the effect of asymmetrical vocal fold anatomy/configuration, which is a well-known cause of pathological voice production and voice nonlinearities [30,31].

The two-mass model was run for 10 s with the standard parameters used in a previous publication [28], an asymmetry coefficient of 0.51, and a time-varying simulated air pressure of the lungs (‘pressure sweep’) ranging from 0 to 2.5 kPa. The time step was 0.05 ms, resulting in a virtual sampling frequency of 20 kHz. For data analysis, the positions of the lower, larger masses were considered.

**Figure 5.**
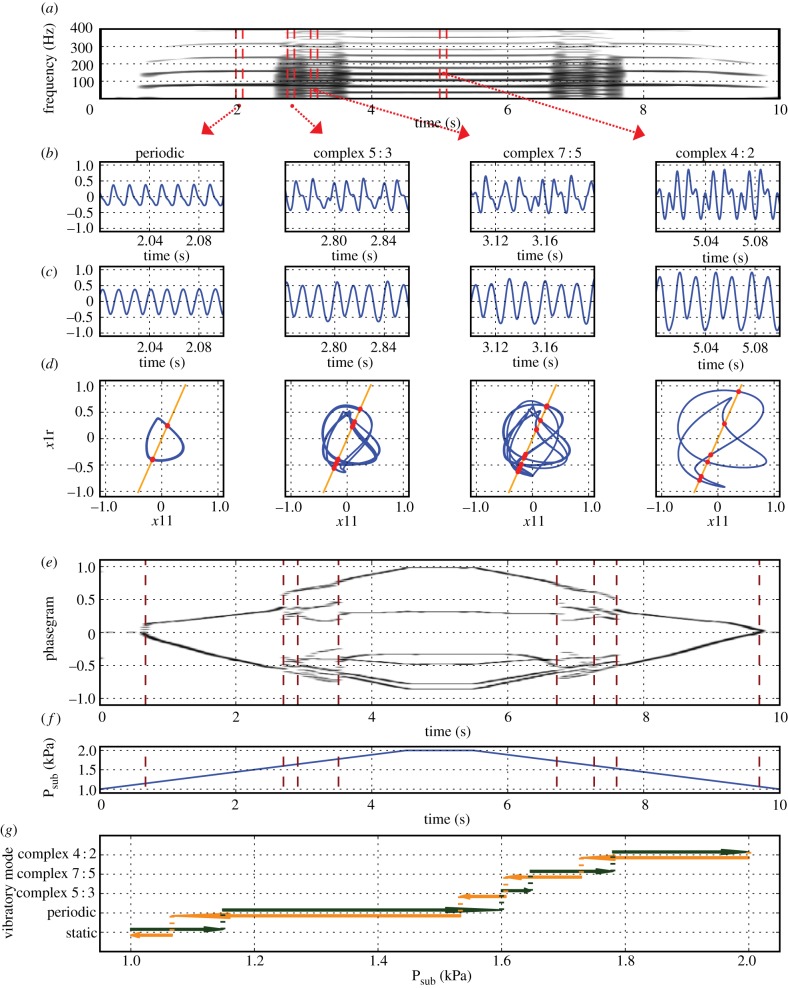
Simulation of vocal fold vibration with a simplified two-mass model (Steinecke/Herzel) during a pressure sweep (0–2.5–0 kPa; *Q* = 0.51). (*a*) Narrow-band spectrogram of the simulated airflow; (*b*) and (*c*) displacement of left (*b*) and right (*c*) lower mass of the model as a function of time, extracted at *t* = 2, *t* = 2.76, *t* = 3.1 and *t* = 5 s. (*d*) Phase portraits from the above signals, created by plotting the time-varying position of the left lower vocal fold mass against that of the right lower mass. A Poincaré section was created at an angle of 0.375 π radians, yielding intersection points with the trajectory (red dots). (*e*) Phasegram: the vertical markers at *t* = 0.67; 2.7; 2.91; 3.51; 6.72; 7.27; 7.6 and 9.7 s represent bifurcations, i.e. changes from one distinct oscillatory regime to another (see text). (*f*) Trace of the time-varying simulated lung pressure used to drive the model. The vertical markers indicate bifurcation events (see above and text). (*g*) Bifurcation diagram, showing the distinct vibratory patterns in relation to lung pressure. Note the hysteresis caused by the direction of lung pressure change (increasing versus decreasing—see text for details).

Following Steinecke & Herzel [28], the time-varying displacements of the model's two lower masses were plotted against each other (figure 5). Four distinct vibratory regimes were seen in the right mass and in the glottal flow (not shown here) for the chosen model parameters: periodic, period doubling and other subharmonic regimes (see also electronic supplementary material, movie S3). Owing to the user-defined asymmetry in the model geometry and mechanical properties, the left mass had a more complex vibratory pattern than the right mass. The right and the left masses are synchronized by ratios of 1 : 1, 5 : 3, 5 : 7 and 4 : 2, respectively, for the four examples shown in figure 5*b*–*d*.

The four vibratory regimes can be clearly distinguished from each other in the phasegram (figure 5*e* and table 1).

**Table 1.** Oscillatory regimes seen in the time series data from the self-oscillating two-mass model simulation of the vocal folds.

oscillatory regime	phasegram features	time (s)
static	no oscillation—no line seen in the phasegram (caused by a slight DC offset in the analysed signals)	0 … 0.67; 9.7 … 10
periodic	gradual amplitude variation—two horizontal lines seen in the phasegram	0.67 … 2.7; 7.6 … 9.7
5 : 3 synchronization	complex vibratory pattern—six horizontal lines seen in phasegram	2.7 … 2.91; 7.27 … 7.6
7 : 5 synchronization	complex vibratory pattern—eight horizontal lines seen in phasegram	2.91 … 3.51; 6.72 … 7.27
4 : 2 synchronization	complex vibratory pattern—two horizontal lines in the upper half of the phasegram (stemming from the period doubling in the right vocal fold mass—figure 5*c*) and four horizontal lines in the lower half (caused by the period-quadrupling seen in the left vocal fold mass—figure 5*b*)	3.51 … 6.72

When relating these four vibratory regimes to the time-varying simulated lung pressure used in the simulation (figure 5*f*), a typical hysteresis effect is seen (figure 5*g*): the model's behaviour was different for the pressure increase (*t* = 0–4 s) versus the decreasing pressure (*t* = 6–10 s). The system tended to stay in its current vibratory regime, and for certain pressure values (1.6–1.68 kPa; 1.71–1.73 and 1.82–1.88 kPa) more than one vibratory regime was possible—see references [31–37] for more detail on hysteresis phenomena in voice. The phonation threshold pressure [38], i.e. the minimum pressure required for the vocal folds to start and stop the vibration (1.17 and 1.08 kPa, respectively), was higher for phonation onset than for phonation offset (representing a subcritical Hopf bifurcation).

### 3.4. Case 4: excised larynx experiment

Excised larynx experiments allow experimental investigation of vocal production under controlled conditions [39]. The larynx (harvested from a freshly deceased individual) is mounted on a vertical air tube, and the vocal folds are adducted (approximated to the sagittal midline). Humidified heated air is blown through the larynx, and the vocal folds exhibit flow-induced self-sustaining oscillation if boundary conditions are properly set. In such a set-up [40, ch. 1], one or more of three basic parameters are usually controlled and varied: air pressure, vocal fold adduction and longitudinal stress in the vocal folds (vocal fold elongation).

For the purpose of this study, an excised larynx of a 6.5-year-old male sika deer (*Cervus nippon*) was examined (figure 4*b*). The experimental set-up has been described in detail elsewhere [41]. Subglottal air pressure was varied in a range of 0–4.1 kPa, as measured with a Keller PR-41X pressure sensor positioned 32 cm upstream from the vocal folds. Pressure data were captured with a LabJack U6 data acquisition card at a sampling rate of 1 kHz. The acoustic signal was recorded using a DPA 4061 omnidirectional microphone positioned 7 cm from the vocal folds.

Vocal fold vibration was monitored by means of EGG, a non-invasive technique that records a physiological correlate of vocal fold vibration during phonation [42–44]—see electronic supplementary material for details concerning the method and the experimental set-up.

**Figure 6.**
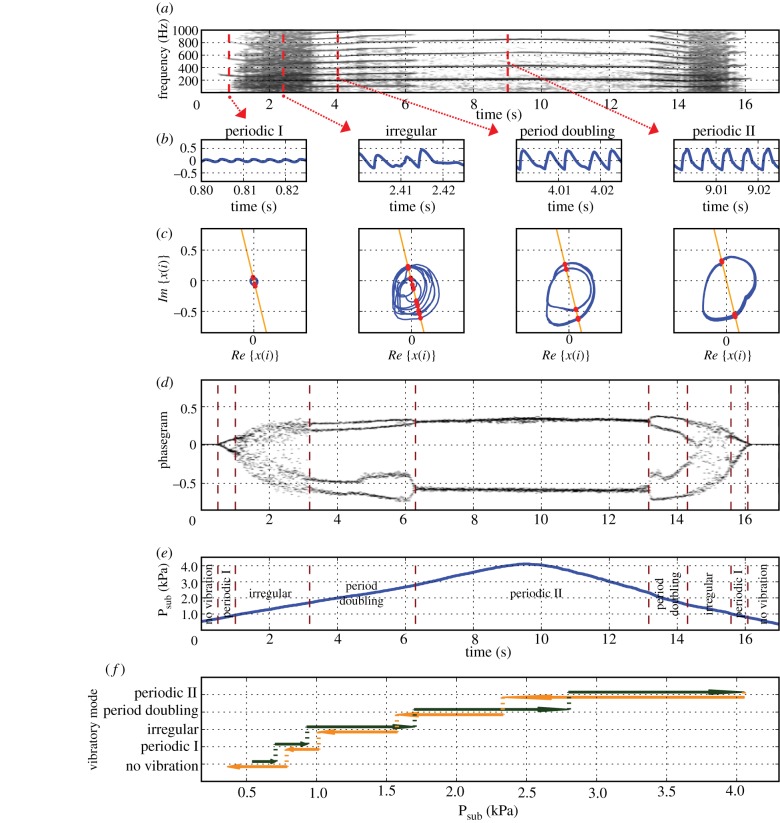
Artificial phonation of an excised sika deer larynx produced during a pressure sweep (0–4.1–0 kPa). (*a*) Narrow-band spectrogram of the electroglottographic (EGG) signal; (*b*) EGG signal, extracted at *t* = 0.725, *t* = 2.3, *t* = 4 and *t* = 10 s. (*c*) Phase portraits from the above signals, created by plotting the real portion of the Hilbert-transformed EGG signal against its imaginary counterpart. A Poincaré section was created at an angle of 0.575 π radians, yielding intersection points with the trajectory (red dots). (*d*) Phasegram: the vertical markers at *t* = 0.48, *t* = 0.98, *t* = 3.18, *t* = 6.20, *t* = 13.14, *t* = 14.24, *t* = 15.59 and *t* = 16.07 s represent bifurcations, i.e. changes from distinct oscillatory regime to another (see text). (*e*) Trace of the time-varying air pressure: the vertical markers indicate bifurcation events (see above and text). (*f*) Bifurcation diagram, showing the distinct vibratory patterns in relation to air pressure. Note the hysteresis caused by the direction of air pressure change (increasing versus decreasing—see text for details).

Analysis of the EGG signal from excised sika deer larynx oscillations (figure 6*a*,*b*) revealed five distinct vibratory regimes: static (no oscillation; not displayed in figure 6*b*), periodic (small amplitude, gradual amplitude variation), irregular (complex non-periodic signal), period doubling and again periodic (stable at larger amplitude). These vibratory regimes can be readily distinguished from each other in the phasegram (table 2 and figure 6*e*).

**Table 2.** Oscillatory regimes observed in the time series data from a excised sika deer larynx.

oscillatory regime	phasegram features	time (s)
static	no oscillation—one horizontal line seen in the phasegram	0 … 0.49; 16.08 … 16.99
periodic I	small amplitude, gradual amplitude variation—two horizontal lines seen in the phasegram	0.49 … 1.00; 15.58 … 16.08
irregular	non-periodic signal—no stable horizontal lines seen in phasegram	1.00 … 3.19; 14.30 … 15.58
period doubling	four horizontal lines seen in phasegram	3.19 … 6.3; 13.16 … 14.30
periodic II	stable large amplitude—two horizontal lines seen in phasegram	6.3 … 13.16

When relating these vibratory regimes to the time-varying simulated lung pressure used in the experiment (figure 6*e*), a hysteresis effect is seen (figure 6*f*), just as in the two-mass model (recall figure 5): the system's behaviour was different during pressure increase (*t* = 0–9.5 s) when compared with during pressure decrease (*t* = 9.5–16.99 s). For certain pressure values (0.93–1.01; 1.57–1.70; 2.33–2.75 kPa), more than one vibratory regime was possible. Contrary to the two-mass model example, the phonation threshold pressure was lower for phonation onset (0.71 kPa) than for phonation offset (0.79 kPa), which is a surprising finding.

### 3.5. Case 5: human voice signal

As for most mammals, human vocalization is created by flow-induced self-sustaining oscillation of the vocal folds. In non-pathological phonation, four basic vibratory modes (called ‘vocal registers’) are observed [21,45]. Of particular importance for both speech and singing are two of these: the so-called chest register and the falsetto register. In chest register, the vocal folds are thickened by the activity of the thyroarytenoid muscle (situated within the vocal folds [46]), thus introducing a vertical phase delay in the lower versus the upper portion of the vocal folds. In general, the duration of vocal fold contact (i.e. the (partial) cessation of air flow) within one oscillatory cycle is longer in chest than in falsetto, resulting in stronger high-frequency harmonics (integer multiples of the fundamental frequency) in the generated acoustic output, and thus a ‘brassier’ sound [47,48]. In falsetto, the thyroarytenoid muscle is relaxed, vertical phase differences are less pronounced, the duration of vocal fold contact is shorter and weaker high-frequency partials are generated, leading to a ‘purer’, more ‘flutey’ sound. In speech, both males and females mainly use the chest register, which is generally lower in fundamental frequency compared with falsetto register. In soft- or high-pitched singing (at a higher fundamental frequency), the falsetto register is used, particularly by females [49].

A 52-year-old semi-professional singer (baritone) sang a sustained note at vowel /a/ near the upper end of his frequency range (pitch of C#4, fundamental frequency *ca* 277 Hz). He was asked to vary vocal intensity from a minimum to a maximum, and back to a minimum, without taking a breath during the exercise. The goal was to perform this manoeuvre on a single musical pitch (i.e. by minimizing changes of fundamental frequency) without introducing abrupt audible changes into the ‘sound colour’ of the generated sound. Please refer to the electronic supplementary material for details on the experimental set-up.

**Figure 7.**
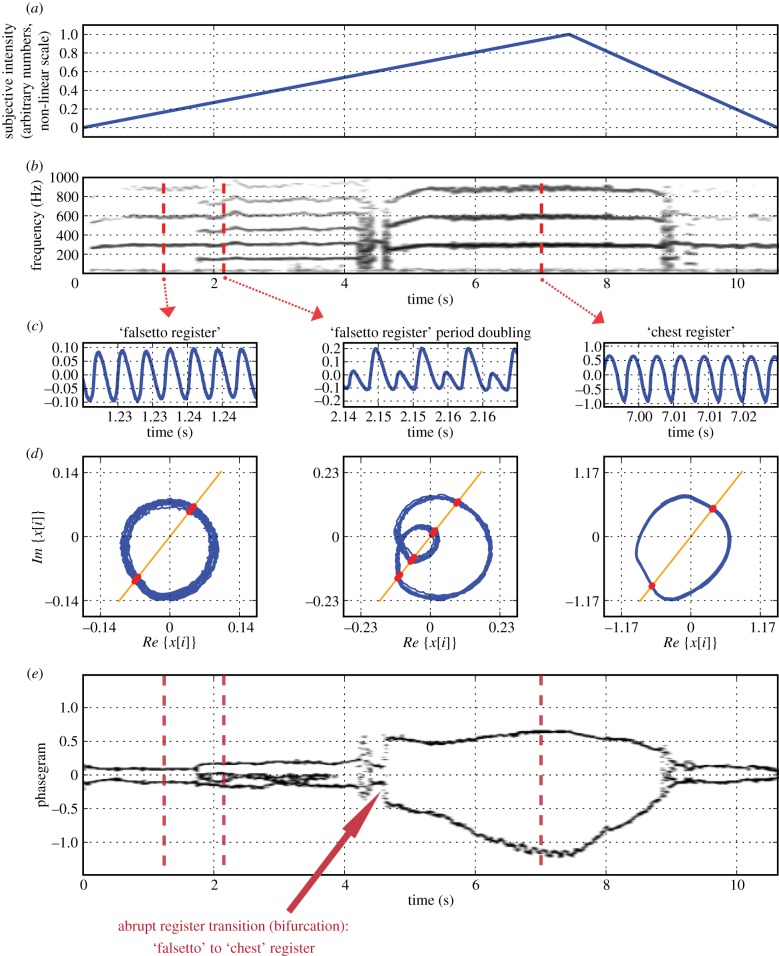
Electroglottographic recording of a 52-year-old singer producing a sustained note (vowel /a/) with intensity variation (soft–loud–soft). (*a*) Singer's attempted intensity of voice production in arbitrary numbers on a nonlinear scale (0, lowest intensity; 1, highest intensity). (*b*) Narrow-band spectrogram of the electrographic (EGG) signal. (*c*) EGG signal, extracted at *t* = 1.22, *t* = 2.14 and *t* = 7 s. (*d*) Phase portraits from the above signals, created by plotting the real portion of the Hilbert-transformed EGG signal against its imaginary counterpart. A Poincaré section was created at an angle of 0.3 π radians, yielding intersection points with the trajectory (red dots). (*e*) Phasegram: the vertical markers at *t* = 1.22, *t* = 2.14 and *t* = 7 s indicate the time instants at which the signals shown in panel (*c*) were extracted.

Human voice production is governed by complex control parameters, over which the singer has only partial and intuitive control. In the example shown in figure 7, the singer's intended intensity of voice production was varied, attempting to keep all other parameters stable. The plotted intensity is a dimensionless quality, expressed on an arbitrary nonlinear scale (0, lowest intensity; 1, highest intensity; figure 7*a*). The spectrogram in figure 7*b* reveals several abrupt transitions, suggesting spontaneous changes in the underlying voice production mechanism, not intended by the singer. They represent unwanted, spontaneous system-level behaviour and violate the traditional aesthetic boundary conditions of classical singing. The findings, corroborated by inspection of the time-domain signal (provided as the audio track in electronic supplementary material, movie S5), are described in table 3.

**Table 3.** Observed system state transitions in the case of an inadequately executed intensity variation manoeuvre in singing.

offset (s)	observed phenomenon
0	periodic
1.75	period doubling
4.2	chaos
4.4	periodic
4.59	chaos
4.63	periodic
8.82	period doubling
9	periodic
9.18	subharmonic regime
9.3	periodic

EGG signals of the most prominent vibratory regimes were extracted at time instants *t* = 1.22, *t* = 2.14 and *t* = 7, and are shown in figure 7*c*. Based on the knowledge gained from previous research [48,50,51], the sound production mechanisms of the three extracted samples can be classified as ‘falsetto register’, ‘falsetto register with period doubling’ and ‘chest register’, respectively. Phase portraits of the three extracted signal portions are shown in figure 7*d*, and the respective positions in time of their Poincaré sections are marked as vertical lines in the resulting phasegram in figure 7*e*. The abrupt transitions between various vocal fold vibratory regimes as observed in the spectrogram (figure 7*b*) are also clearly reflected in the phasegram. The arrow in figure 7*e* marks the position of an abrupt transition from falsetto to chest register, occurring over an interval of *ca* 18 ms (five vibratory cycles). At that instant of time, the fundamental frequency dropped abruptly from 320 to 242 Hz (as seen in the spectrogram, figure 7*b*).

## 4. Discussion

Phasegrams provide a new tool to visualize and gain insights into system dynamics, particularly for nonlinear dynamical systems in which the vibratory regime changes over time. In a phasegram, the intuitiveness and accessibility of the spectrogram is combined with the more detailed information on system dynamics provided by phase space analysis. Basically, a phasegram is an empirically derived bifurcation diagram in time. However, in contrast to the ‘traditional’ bifurcation map approach, underlying system parameters do not need to be known for phasegram generation. This is particularly useful in situations where only an output signal of the (bio)physical system of interest is available.

The creation of phasegrams was motivated by the goal of visualizing a system's dynamics over time in a single two-dimensional graph. To accomplish this, considerable data reduction is required: the creation of consecutive time-varying two-dimensional phase portraits by means of attractor reconstruction results in three-dimensional data. A further reduction is achieved by performing Poincaré sections through the generated phase portraits, thus reducing the data to two dimensions: in a phasegram, time is displayed on the *x*-axis, and the time-varying intersection points through the Poincaré sections are displayed on the *y*-axis (as an additional feature, the frequency of occurrence of the intersections is reflected as colour intensity along a virtual *z*-axis). The resulting two-dimensional graph provides intuitive insights into the analysed system's dynamic behaviour. Phasegram figures are, due to their two-dimensional nature, expected to be particularly useful in printed publications.

For an ideal Poincaré section orientation (see 2.3), the dynamics of the analysed system can be assessed via the number and stability of horizontal lines in the phasegram.

— One single line: static (no oscillation).
— 2*n* locally stable lines: limit cycle, with optional subharmonics if *n* > 1, i.e. periodic oscillation for two lines, period doubling for four lines, subharmonic regime with period tripling [52] for six lines, etc. If the analysed nearly periodic signal contains substantial spectral energy above the fundamental (i.e. harmonics), additional trajectory contours are introduced into the phase portraits, their Poincaré sections, and consequently into the generated phasegrams. In such cases, low-pass filtering the analysed signal may be considered.
— Multiple, locally unstable lines: irregularity caused by either changing system parameters; a quasi-periodic signal with more than two individual sinusoids whose frequencies are not related to each other by integer ratios; or chaos.

In the range of examples considered here, we have found the phasegram to be a useful tool for classification of (bio)physical signals into three main categories: periodic oscillation, subharmonic regimes and chaos. This potential needs to be verified in further studies and other systems.

Just as in other analysis and visualization techniques (e.g. spectrograms), the appearance of a phasegram (and hence the information content provided by the technique) depends on the selection of various parameter values—see electronic supplementary material. As is the case in any analysis of experimental time-series data, noise in the measurement may introduce unwanted effects: when analysing signals with a low signal-to-noise ratio, the generated phase portraits and hence the phasegram itself can adapt a ‘noisy’ appearance.

To generate a phasegram, Poincaré sections are needed that intersect with a significant portion of the trajectories in phase space, in order to reveal the governing dynamics of the system. Such an approach cannot guarantee transversality of all Poincaré sections. In the light of this issue, the applicability of the phasegram method depends on the global structure of the dynamics of the analysed time series. However, for many practical purposes any Poincaré section—regardless of transversality—will produce useful insights into system dynamics. Oscillatory dynamics, in general, and acoustic and other voice-related physiological data, in particular, are especially suited for phasegram analysis: These signals repeatedly cross zero by construction (e.g. due to pressure fluctuations), and the origin of the phase space is used as the anchor point for the Poincaré sections. In other cases, where these zero-crossings are not present, a simple removal of the DC offset or trend found in the data may facilitate a phasegram representation.

In the past, several methods for two-dimensional space–time visualization have been introduced, such as, for example, phase space diagrams stemming from numerical solutions of equation systems [16], phase space attractor reconstruction [12,13], recurrence plots [53] or coarse-grained embeddings of time series [54–56]. These tools are very helpful in visualizing the dynamics of a system over a certain (and typically short) period of time, by analysing the underlying time-series data (consisting of a certain number of samples) and generating one graph for that particular time window. However, such representations fail to appropriately illustrate changes of system state (e.g. from periodic oscillation to period doubling or chaos). In particular, visualization tools that do not map time onto either the ordinate or the abscissa are unable to pinpoint within a single graph the exact points in time at which bifurcations occur. This is particularly crucial for signals that are characterized by frequent changes of oscillatory regime, as is found frequently in time-series stemming from biosystems or acoustic data (see figure 7*e* for an example).

To overcome this limitation, we developed the phasegram, based on attractor reconstruction and Poincaré sections, which are already well-established visualization techniques. In contrast to conventional sliding-window analysis, such as the spectrogram [57,58], the Wigner–Ville distribution [59] or the scaleogram [60], the phasegram offers direct insights into the dynamics of the analysed system, because it is generated from the signal's embedded attractor. No advance knowledge of periodicity or fundamental frequency is required for phasegram generation, which makes the tool superior to previously presented sliding-window visualization approaches such as the time-varying sequence of local maxima [24] or EGG wavegrams [11].

Although the phasegram is not equipped to measure the dimensionality of complex attractors, the examples shown in this manuscript suggest that the new method is very well suited to visualize the temporal evolution of a dynamical system's attractors, from very simple to complex. Two embedding dimensions are sufficient to fully describe (nearly) periodic signals and to evaluate the signal's divergence from such a periodic pattern. In this respect, phasegrams offer an empirical basis for the decision of whether the signal's fundamental frequency can be calculated, or whether (computer-aided) periodicity analysis will give spurious results due to the occurrence of subharmonics, quasi-periodicity or other forms of irregularity.

In this paper, the application of phasegrams for system dynamics visualization has been exemplified for voice production. However, the technique is by no means limited to vocal applications. As our initial logistic map and Lorenz system examples show, phasegrams can usefully be applied in any branch of physics, biology, chemistry, economics or other fields where nonlinear systems are analysed and an intuitive indication of system dynamics and how they change over time is desired.

## Endnotes

1 To make the contents of this manuscript accessible to researchers from disciplines other than physics or mathematics, we include some tutorial-style background information on these signal types as electronic supplementary material.

2 A signal is classified as non-stationary if its probability distributions are time-dependent. Non-stationarity is given if a time-series data have a mean, variance or covariance that changes over time. Non-stationary features manifest themselves as trends, cycles or random walks. Most biological signals (but also, e.g. economic time-series or meteorological data) can be approximated as stationary on a short scale, but are non-stationary when observed over a longer time span. Non-stationarity in (bio)physical systems arises when system parameters change, e.g. the neurally controlled tension of the vocal folds in voice production.

3 Alternatively termed ‘phase space plot’.

4 The DC offset is the arithmetic mean of a time-series data.

5 The phasegram software is available online at the first author's website at http://www.christian-herbst.org/phasegrams/.
